# Therapeutic Potential of *Lindera obtusiloba*: Focus on Antioxidative and Pharmacological Properties

**DOI:** 10.3390/plants9121765

**Published:** 2020-12-13

**Authors:** Md Ezazul Haque, Shofiul Azam, Rengasamy Balakrishnan, Mahbuba Akther, In-Su Kim

**Affiliations:** 1 Department of Applied Life Science, Graduate School, Konkuk University, Chungju 27478, Korea; mdezazulhaque@yahoo.com (M.E.H.); shofiul_azam@hotmail.com (S.A.); rmbalabio@gmail.com (R.B.); smritymahbuba@gmail.com (M.A.); 2 Department of Biotechnology, College of Biomedical and Health Science, Research Institute of Inflammatory Disease (RID), Konkuk University, Chungju 27478, Korea

**Keywords:** *Lindera obtusiloba*, antioxidant, oxidative stress, anti-cancer, anti-inflammatory, anti-platelet, anti-allergic

## Abstract

*Lindera obtusiloba* (LO) BLUME from the genus *Lindera* (Lauraceae) is a medicinal herb traditionally used in Southeast Asian countries. Indigenously, extracts of different parts of the plant have been used to improve blood circulation and treat allergy, inflammation, rheumatism, and liver diseases. LO is a rich source of therapeutically beneficial antioxidative phytochemicals, such as flavonoids, butenolides, lignans and neolignans. Moreover, recent studies have unravelled the pharmacological properties of several newly found active constituents of LO, such as anti-inflammatory antioxidants (+)-syringaresinol, linderin A, anti-atherosclerotic antioxidant (+)-episesamin, anti-melanogenic antioxidants quercitrin and afzelin, cytotoxic 2-(1-methoxy-11-dodecenyl)-penta-2,4-dien-4-olide, (2*Z*,3*S*,4*S*)-2-(11-dodecenylidene)-3-hydroxy-4-methyl butanolide, anti-allergic koaburaside, (6-hydroxyphenyl)-1-O-beta-d-glucopyranoside and 2,6-dimethoxy-4-hydroxyphenyl-1-O-beta-d-glucopyranoside and the antiplatelet-activity compound Secolincomolide A. These findings demonstrate that LO can be a potential source of antioxidants and other prospective therapeutically active constituents that can lead to the development of oxidative stress-mediated diseases, such as cardiovascular disorders, neurodegenerative disorders, allergies, inflammation, hepatotoxicity, and cancer. Here, the antioxidant properties of different species of *Lindera* genus are discussed briefly. The traditional use, phytochemistry, antioxidative and pharmacological properties of LO are also considered to help researchers screen potential lead compounds and design and develop future therapeutic agents to treat oxidative stress-mediated disorders.

## 1. Introduction

*Lindera,* a core genus containing more than 100 species, is a member of the Litseeae tribe under the Lauraceae family. Plants of the *Lindera* genus are widely distributed all over the world, particularly in the tropical, subtropical and temperate regions of Asia and midwestern America [1]. Plants from the *Lindera* genus are considered a rich source of essential oils and are often used in the production of aromatic cosmetic products such as soap and lubricants for their elegant fragrance [2]. Most importantly, throughout history, many *Lindera* plants have been used in traditional medicine for their healing and curing capabilities for several health-related implications, such as pain, cold, urinary tract disorders, rheumatoid arthritis, gastric ulcer, abdominal pain, cholera, and beriberi [3,4]. Surprisingly, plants of the *Lindera* genus have been reported to produce almost 350 chemical constituents, which mostly belong to sesquiterpenoids, alkaloids, phenylpropanoids, butanolides, lucidones, flavonoids, etc. [2,3]. Studies have shown that *Lindera* genus plants possess anti-cancer, anti-inflammatory, antihypertensive, and analgesic properties. Moreover, several species of this genus have been reported as a rich source of antioxidants, including *Lindera aggregata* (Sims) Kosterm, *Lindera erythrocarpa* Makino, and *Lindera pulcherrima* (Nees) Hook. f. [5,6,7].

The human body is consistently fighting oxidative attacks from reactive oxygen and nitrogen species (RONS) using a complex antioxidant defence system to maintain the pro-oxidant–antioxidant balance [8]. Imbalance in the cellular antioxidant defence machinery can result from several factors, including ageing and environmental toxins [9]. However, chronic imbalance in the cellular oxidative state may lead to several age- or un-related disorders such as neurodegenerative, cardiovascular, chronic obstructive pulmonary, and chronic kidney diseases as well as cancer [10]. Reactive oxygen participates in the high-energy electron transfer mechanism and the production of adenosine-5-triphosphate (ATP) through oxidative phosphorylation [8]. Imbalance in the production of RONS and antioxidant defence in the cell leads to oxidative stress and attack of many biological molecules such as proteins, lipids, or DNA [10]. Several redox-sensitive transcriptional factors and enzymes such as nuclear factor-erythroid 2-related factor (Nrf-2) and activate antioxidant response element (ARE) have been implicated in maintaining cellular oxidative homeostasis [11]. Activation of ARE results in the up-regulation of several antioxidant enzymes, such as heme oxygenase-1 (HO-1), which catalyzes the oxidative degradation of heme into bilirubin and free iron and NAD(P)H quinine oxidoreductase 1 (NQO1); this latter enzyme regulates reactive oxygen species (ROS) generation by reducing quinones to hydroquinones [12]. Therefore, Nrf-2/HO-1/NQO1 are involved in maintaining the balance of oxidative stress, thus protecting cells and tissues from oxidative damage [11,12].

Antioxidants are used for the prevention and treatment of oxidative stress-induced diseases, and plants are used as natural sources of antioxidant compounds. Natural antioxidants can decrease the generation of RONS, scavenge free radicals and prevent the lipid peroxidation process [13]. Plant-derived antioxidants or plants rich with antioxidant compounds have been used for thousands of years for treating various pathological conditions associated with ageing and oxidative stress [14].

*Lindera obtusiloba* Blume (LO), a member of the genus *Lindera*, is widely distributed in northeast Asia. The use of LO as a traditional medicine for the treatment of improvement of blood circulation, inflammation fever, abdominal pain, etc., is well documented. Both extracts of the different parts of LO and its bioactive compounds have been reported to contain numerous antioxidative compounds, such as polyphenols, lignans, neolignans, flavonoids, and butenolides. The neuroprotective, cytotoxic, anti-inflammatory, anti-allergic, and antithrombotic properties of these bioactive compounds have been reported [15,16,17,18]. Therefore, LO presents a rich source of bioactive compounds, particularly antioxidants that have been investigated in many diseases associated with oxidative stress. Still, many new antioxidant compounds are being isolated from the extract of different parts of LO and need to be investigated for their antioxidant and pharmacological activity. Here, we briefly discuss the antioxidant properties of the genus *Lindera* and focus on their traditional and ethnopharmacological use, bioactive compounds isolated from LO extracts and their antioxidant and pharmacological properties. Moreover, we mention many newly isolated or detected compounds from LO extracts that could prove beneficial to developing new therapeutic agents to treat oxidative stress-associated disorders such as cardiovascular disorders, neurodegenerative disorders, allergy, inflammation, and cancer.

## 2. Genus *Lindera* and Antioxidant Properties of Its Plants

Throughout history, plant extracts have been used as remedies for several pathological conditions associated with oxidative stress [19,20]. The secondary metabolites isolated from the plants of the genus *Lindera* consist of several types of phytochemicals, namely sesquiterpenoids (Linderagalactone E, furanodienone, curzerenone), alkaloids (*N*-methyllaurotetanine, (+)-isoboldine), flavonoids (Quercetin-3-O-α-l-rhamnopyranoside, quercitrin, (−)-epicatechin, Afzelin), phenylpropanoids, butanolides ((2*Z*,3*S*,4*S*)-2-(11-dodecenylidene)-3-hydroxy-4-methyl butanolide and (2*E*,3*R*,4*R*)-2-(11-dodecenylidene)-3-hydroxy-4-methoxy-4-methyl butanolide), lucidones, etc. [2]. These secondary metabolites and bioactive compounds isolated from the plants of the *Lindera* genus have been reported to possess various pharmacological activities (e.g., antioxidant, anti-allergic, antimelanogenic, cytoprotective, anti-inflammatory, and antitumour) both in vitro and in vivo (Table 1) [5,21,22,23,24,25].

The *Lindera* genus is part of the family Lauraceae, which is widely distributed in tropical, sub-alpine and temperate regions of the Asian and American continents, with approximately 80 to 100 species [26,27]. Among them, *Lindera aggregata* (Sims) Kosterm*, Lindera glauca* (Siebold et Zucc.) Blume*, Lindera neesiana* (Wall. ex Nees) Kurz, *Lindera pulcherrima* (Nees) Hook. F., *Lindera benzoin* (L.) Blume*, Lindera chunii* Merr., *Lindera obtusiloba* Blume*, Lindera angustifolia* W.C. Cheng, and *Lindera reflexa* Hemsl. species are used as traditional medicines for their therapeutic effect on whitening, hepatitis C, hepatotoxicity, anti-cancer, antibacterial, antiproliferative, endothelial dysfunction, neuroprotection, antifibrotic, and effects on post-ischemic myocardial dysfunction [5,6,7,28]. However, due to the abundance of antioxidant compounds in *Lindera* genus plants, this genus can be considered a potential source of natural compounds that can be used for the development of therapeutic agents to treat oxidative stress-induced diseases.

*L. aggregata* (LA) is widely used as a tea in China and Southeast Asian countries. Both ethanolic and water extract of different parts of *L. aggregate* have been shown to possess antioxidant activities [21]. Water and EtOH extract of *Lindera* radix and the dried root of LA have been reported to decrease methane dicarboxylic aldehyde (MDA) and superoxide dismutase (SOD) levels and the expression levels of nuclear factor (NF-κB), tumour necrosis factor (TNF-α) and interleukin (IL-1β) in alcoholic liver injury. Further, the extract improved the histopathological status and decreased the serum levels of alanine aminotransferase (ALT), aspartate aminotransferase (AST), triglyceride (TG), total cholesterol (TC), and MDA and NF-κB, TNF-α, and IL-1β in liver tissues [21]. EtOH extract of LA leaves also showed free radical scavenging activity in a 2,2-diphenyl-1-picryl-hydrazyl-hydrate (DPPH) assay. Eleven polyphenols were identified in this extract by HPLC. A higher amount of quercetin-3-O-α-l-rhamnoside was detected in the extract and showed strong antioxidant capacities. Two alkaloids—linderaggredin C (3), (+)-*N*-methyllaurotetanine and (+)-isoboldine—isolated from the extract showed significant inhibition of superoxide anion generation in human neutrophils [22,23]. Furthermore, linderanean, which is an active compound isolated from LA root, increased activation of the Nrf-2 pathway in INS-1 cells and protected it from streptozotocin-induced apoptosis [24]. Five sesquiterpene lactones—lindera, galactone E, linderane, hydroxylindestenolide, and linderalactone—were isolated from the roots of LA and showed hepatoprotective activity against H_2_O_2_-induced oxidative damage on HepG2 cells [25]. Quercetin, quercetin-3-O-α-L-rhamnopyranoside, and quercetin-3-O-α-l-rhamnoside were found in high concentrations in the LA leaves, demonstrating free radical scavenging activity and modulation of the Nrf-2 pathway [5,22,29]. Overall, most of the compounds identified in the extracts of LA were associated with a higher level of antioxidant activities in different assays; hence, LA could be a potential source of antioxidant compounds and should be further studied for its therapeutic possibilities.

*Moreover, LA*, has long been used as a traditional medicine for rheumatic, cardiac and renal diseases in Japan and other countries. The water extract of its roots has been found to scavenge free radicals in a DPPH assay, and the leaf extract showed ROS, reactive nitrogen species (RNS), and superoxide anion scavenging activity as well as inhibition of lipid peroxidation and protein oxidation [6]. In an isolated rat heart, LA root extract protected against post-ischemic left ventricular dysfunction through scavenging hydroxyl radicals and opening the mitochondrial potassium ATP (K_ATP_) channels [6,30]. Lindenenyl acetate, a compound isolated from the MeOH extract of the roots of LA, was reported to possess strong neuroprotective activity against glutamate-induced oxidative injury in hippocampal neuronal cells, most likely via extracellular signal-regulated kinase (ERK) pathway-Nrf-2/ARE-dependent HO-1 expression. Further, lindenenyl acetate increased the expression of HO-1, accumulation of Nrf2 and increased the promoter activity of ARE in mouse hippocampal HT22 cells [31]. Overall, both extract and bioactive compounds isolated from LA showed strong neuroprotective and cardioprotective activity via modulation of the cellular oxidative imbalance.

*L. erythrocarpa* (LE) is a widely distributed shrub in China, Japan, Korea, and Taiwan. Its dried fruits, which are also referred to as red mountain pepper, are used in folk medicine for indigestion and pain [32]. Lucidone, a cyclopentenedione isolated from the fruits of LE, has demonstrated significant protective abilities against free-radical and inflammation stimulator 2,2’-azobis (2-amidinopropane) dihydrochloride (AAPH)-induced oxidative stress in human keratinocyte cells (HaCaT) through up-regulating HO-1/Nrf-2 gene expression and down-regulating the NF-κB signalling pathway [33]. In addition, lucidone suppressed hepatitis C viral replication by induction of Nrf-2-Mediated HO-1 in Ava5 cells [34]. Moreover, among eight compounds isolated from the methanol fraction of LE*,* (−)-epicatechin, avicularin, and quercitrin prevented apoptotic cell death of H9c2 cardiomyocytes treated with buthionine-[S,R]-sulfoximine. These compounds also reduced the propidium iodide uptake by these cells and dose-dependently decreased the release of lactose dehydrogenase (LDH) [28]. Therefore, these three compounds provide a potential lead compound for the development of antioxidative, cardioprotective agents that can be used as anti-viral or cardioprotective agents.

*L. pulcherrima* (Nees.) Benth. (LP), also termed an evergreen shrub, is distributed in temperate Himalayan regions, and is used as a medicinal plant. The leaves and bark are used as a spice for the remedy of cold, fever, and cough. In an in vitro study, the antioxidant activity of the essential oils of LP leaf was assessed by DPPH radical scavenging and inhibition of lipid peroxidation. The essential oils of LP leaf showed potent free radical scavenging activity and inhibition of lipid peroxidation. In another study, two constituents—furanodienone and curzerenone—of the essential oils of LP leaf were investigated for free radical scavenging activity in a DPPH assay and inhibition of lipid peroxidation. These oil constituents showed the same inhibition of lipid peroxidation and free radical scavenging activity [7,35]. These findings suggest that the leaf extract of LP and its constituents have high potency for free radical scavenging and inhibition of lipid peroxidation.

*L. glauca* (LG), another species of the *Lindera* genus, has been reported to possess free radical scavenging activity and can inhibit lipid peroxidation activity. The water and EtOH extracts of LG stem increased cell viability and reduced ROS generation in tert-butyl hydroperoxide-induced oxidative stress in Chang cells. In addition, it also increased the activities of catalase, glutathione peroxidase, glutathione S-transferase, and expression of the superoxide dismutase gene of zebrafish against oxidative stress [36]. Further, ethanolic extract of LG stem and root showed free radical scavenging, nitrite scavenging, and reducing power activities. The polyphenolic content of the LG extract was 70.99 ± 1.88 μg/TAE μg. The LG extract showed high DPPH radical scavenging activity, nitrite scavenging activity and reducing power activities. In addition, stem and root extracts were found to possess high antiproliferative activities in HT-29 and HCT116 cells [37]. Moreover, eight flavonoids isolated from LG—lindeglaucol, lindeglaucone, cilicicone B, tamarixetin 3-O-α-l-rhamnoside, procyanidin A2, cinnamtannin B, cinnamtannin D1, and procyanidin A1—were tested for their inhibition of low-density lipoprotein oxidation; only four of them—procyanidin A2, cinnamtannin B1, cinnamtannin D1, and procyanidin A1—showed strong low-density lipoprotein (LDL) oxidation inhibitory activities [38].

Another species of the genus *Lindera*, i.e., *L. neesiana* (Wall. ex Nees) Kurz (LN) has been reported to possess antioxidant, anti-inflammatory, and neuroprotective activities. Treatment with both water and EtOH extract of LN was found to reduce the production of NO, pro-inflammatory cytokines and iNOS and COX-2 production in lipopolysaccharide (LPS)-stimulated BV-2 cells. Furthermore, LN extract increased the phosphorylation of ERK, p38 and c-Jun N-terminal kinase (JNK) and decreased the activation of microglia cells. The water extract of LN fruit increased the secretion of Nrf-2 in N2a cells and inhibited LDH release in H_2_O_2_-stimulated BV-2 cells [39]. In another study, five kaempferol glycosides—kaempferol 3-O-β-glucopyranosyl(1→2)-[α-rhamnopyranosyl-(1→6)]-β-glucopyranoside-7-O-α-rhamnopyranoside, kaempferol 3-O-sophoroside, kaempferol 3-O-β-glucopyranosyl-(1→2)-[α-rhamnopyranosyl (1→6)]-β-glucopyranoside, kaempferol 3-O-β-glucopyranosyl(1→2)-α-rhamnopyranoside-7-O-α-rhamnopyranoside, and kaempferol 3-O-α-rhamnopyranoside—isolated from 60% EtOH extract of LN leaves and twigs showed moderate scavenging activities on DPPH radicals and potent pancreatic lipase inhibitory activity [40]. These findings suggest that LN is a rich source of potent antioxidants, which show neuroprotection and anti-inflammatory activity.

Several other species of the *Lindera* genus, such as *L. reflexa, L. fruiticosa, L. angustifolia, L. oxyphila,* and *L. umbrellata* have been reported to possess antioxidant activity in separate studies [41,42,43,44,45,46]. Hence, this genus represents a natural source of highly active antioxidant compounds that can scavenge free radicals and inhibit lipid peroxidation. Both extracts and bioactive compounds of this genus can modulate several oxidative pathways, including Nrf-2/HO-1, ERK, JNK, mitogen-activated protein kinase (MAPK), and ARE that are involved in oxidative stress-mediated cell death, cell proliferation, inflammation, etc.

## 3. Ethnomedicinal Use of *Lindera obtusiloba*

*Lindera obtusiloba* Blume is ubiquitously distributed in the north and southeast parts of Asia and has been used in traditional Chinese, Korean, and Japanese medicine over centuries [52,53]. In Korea and China, it is traditionally used for restoring blood stasis and inflammatory disorders [54]. The leaves of LO are traditionally consumed as both tea and food [55]. The consumable aqueous extract of LO demonstrated significant physiological beneficial effects, such as the inhibition of adipogenesis [56]. Further, in Korean traditional medicine, its leaf or branch extracts are widely used to treat liver diseases and for improving blood circulation, insomnia, and anxiety [57]. The young leaves of LO are fried and traditionally used as a Buddhist ceremonial dish. Furthermore, the oil extracted from LO is used as hair oil in some cultures [58]. The barks of LO are used to treat rheumatism in Chinese medicinal practice by heating the bark under the patient’s knee [59].

## 4. Bioactive Compounds of *Lindera obtusiloba*

**Figure 1:**
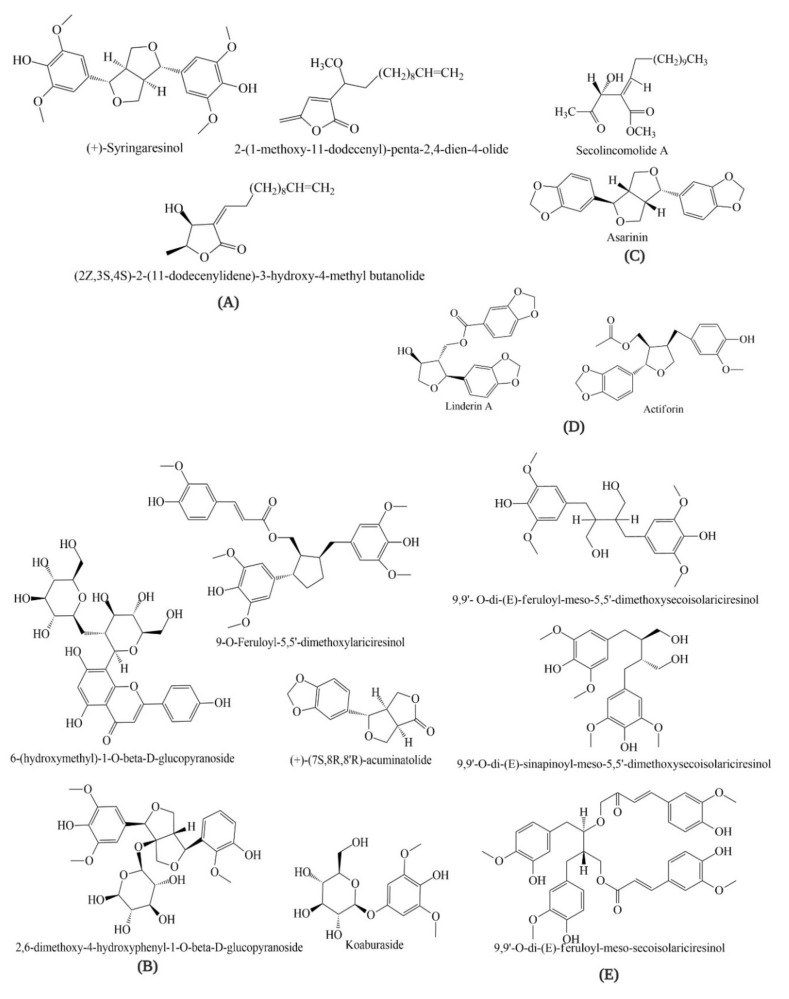
Structures of chemical constituents of *Lindera obtusiloba;* Groups (**A**). Cytotoxic; (**B**). Anti-histamine; (**C**). Antiplatelet; (**D**). Anti-inflammatory; (**E**). Neuroprotective.

There is an array of evidence suggesting that LO is a potential source of antioxidant compounds. Hong et al. (2013) investigated quercitrin (62.9%) and afzelin (22.0%) in a 70% ethanolic extract of LO. Choi et al. (2013) isolated eight phenolic glycosides—tachioside, isotachioside, koaburaside, 2,6-dimethoxy-4-hydroxyphenyl-1-O-beta-d-glucopyranoside, 4,6-dihydroxy-2-methoxyphenyl-1-O-beta-d-glucopyranoside, a mixture of erigeside C and salidroside, and 6-hydroxyphenyl)-1-O-beta-d-glucopyranoside—from the stems of LO and investigated their anti-allergic inflammatory activities [60,61]. Seven neolignans—linderin A, (+)-xanthoxyol, pluviatilol, actiforin, (+)-syringaresinol, (+)-(7*S*,8*R*,8′*R*)-acuminatolide and (+)-9′-O-trans-feruloyl-5,5′-dimethoxylariciresinol—isolated from the stem extract of LO were investigated for their anti-allergic inflammatory effects [62]. Moreover, three new butanolides—2-(1-methoxy-11-dodecenyl)-penta-2,4-dien-4-olide, (2*Z*,3*S*,4*S*)-2-(11-dodecenylidene)-3-hydroxy-4-methyl butanolide and (2*E*,3*R*,4*R*)-2-(11-dodecenylidene)-3-hydroxy-4-methoxy-4-methyl butanolide—from the stems of LO have been reported by Kwon et al. (2000) [63]. The constituents of essential oils from LO leaves, mesocarps, seeds, and barks ware detected by different chromatographic methods. GC-MS analysis of the extract of LO bark detected α-cadinol (11.8%), hedycaryol (9.8%), α-eudesmol (9.7%), caryophyllene (6.4%), T-cadinol (6.2%), terpinolene (5.7%), eudesmol (5.1%), α-elemene (4.8%), cadinene (4.3%), elemene (4.0%), etc [59]. Nil et al. (1983) reported the major oil components of LO; myrcene (20.60%), α-humulene (21.45%), humulol (6.03%) and bornyl acetate (5.06%) in mesocarp; 5-dodecanolide (15.29%), lauric acid (8.74%), bornyl acetate (5.01%) and cis-4-dode-cenoic acid (4.07%) in seed; caryophyllene (7.37%), elemol (5.06%), and unidentified sesquiterpene oxidated compound (7.96%) in leaf [64]. However, many compounds have not been further studied regarding their antioxidant activities. Therefore, these compounds can be considered as potential candidates for further pharmacological studies of different disease models (Figure 1).

## 5. Pharmacological Properties of *Lindera obtusiloba*

### 5.1. Anti-Allergic and Anti-Inflammatory Activities

**Figure 2:**
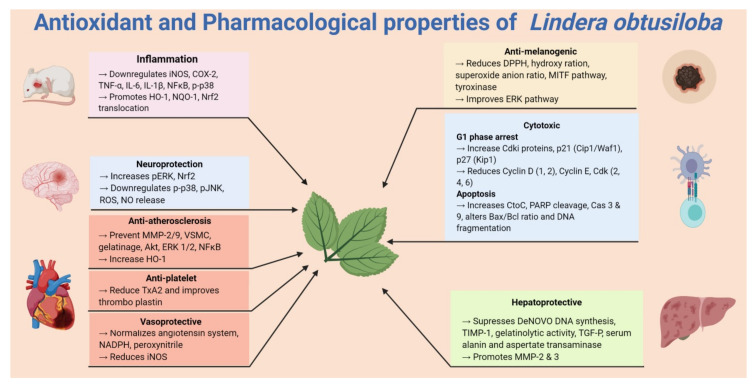
Antioxidative and pharmacological properties of *Lindera obtusiloba*.

Recently, LO has been reported to attenuate oxidative stress and airway inflammation in ovalbumin-challenged (OVA) asthma mice and TNF-α-stimulated NCI-H292 cells (Figure 2). In TNF-α-stimulated NCI-H292 cells, LO leaf extract (LOLE) (~100 μg/mL) increased HO-1 and NAD(P)H quinine oxidoreductase 1 (NQO1) expression and promoted the translocation of Nrf-2 into the nucleus, thus effectively suppressing ROS, NO and lipid peroxidation [15]. In the animal model LOLE effectively suppressed ROS and NO in TNF-α-stimulated NCI-H292 cells. Overall, the study showed the potential effect of LOLE in both in vivo and in vitro analyses of allergic asthma [15]. In another study of ovalbumin (OVA)-challenged mice, treatment with LOLE (~100 mg/kg) effectively inhibited the phosphorylation of MAPKs and NF-κB and suppressed activator protein AP-1, Th2 cytokines and mucin 5AC (MUC5AC) in the lung tissues of mice. LOLE also reduced the expression of inflammatory cytokines and NF-κB activation. Further study showed that LOLE effectively suppressed the ROS and NO levels, and thus markedly suppressed the inflammation of airways and mucus production of the OVA-challenged mice [15].

Eight phenolic glycosides compounds—tachioside, isotachioside, koaburaside, 2,6-dimethoxy-4-hydroxyphenyl-1-O-beta-d-glucopyranoside, 4,6-dihydroxy-2-methoxyphenyl-1-O-beta-d-glucopyranoside, a mixture of erigeside C and salidroside, and 6-hydroxyphenyl)-1-O-beta-d-glucopyranoside—isolated from the stem extracts of LO (LOSE) showed anti-allergic inflammatory activities. Among the eight compounds, koaburaside, (6-hydroxyphenyl)-1-O-beta-d-glucopyranoside and 2,6-dimethoxy-4-hydroxyphenyl-1-O-beta-d-glucopyranoside suppressed the release of histamine from mast cells as compared with gallic acid, which was used as a positive control. In particular, (6-hydroxyphenyl)-1-O-beta-d-glucopyranoside attenuated the gene expressions of the pro-inflammatory cytokines TNF-α and IL-6 in human mast cells [60]. Similarly, seven neolignans, including one new compound linderin A isolated from LOSE, were investigated for their anti-allergic inflammatory effects on human mast HMC-1 cells. Other lignans investigated were (+)-xanthoxyol, pluviatilol, actiforin, (+)-syringaresinol, (+)-(7S,8R,8′R)-acuminatolide and (+)-9′-O-trans-feruloyl-5,5′-dimethoxylariciresinol. All compounds except (+)-xanthoxyol and (+)-syringaresinol inhibited the release of histamine, whereas linderin A and actiforin suppressed the expressions of TNF-α and IL-6 pro-inflammatory cytokines [62]. In another study, (+)-syringaresinol, one of the constituents of LOSE, was reported to possess an anti-inflammatory effect on carrageenan-induced hind paw oedema in mice. Moreover, treatment with (+)-syringaresinol successfully inhibited the protein expression of iNOS, COX-2 and NF-κB and mRNA expression of iNOS, COX-2, TNF-α, IL-1β, and IL-6. It also reduced LPS-induced the release of NO, PGE2, TNF-α, IL-1β, and IL-6 in a dose-dependent manner [65]. These results demonstrate that compounds isolated from LO show strong anti-allergic and anti-inflammatory activity and can, therefore, be further developed into effective therapeutic agents with modification.

### 5.2. Antiplatelet Activity

One study demonstrated the effect of LO leaf extract (LOLE) platelet activities, coagulation, and thromboembolism in in vitro and ex vivo experiments (Figure 2). In rat platelet, LOLE significantly inhibited collagen-induced thromboxane A2 (TXA2) production. A mixture of collagen and epinephrine induced pulmonary thromboembolism in mice. Oral administration of LOLE significantly altered the activated partial thromboplastin time (aPTT) but not prothrombin time (PT). However, the results demonstrate that LOLE extract possesses antithrombotic effects that might be due to its antiplatelet activities [54]. In addition to concentration-dependent inhibition collagen- and ADP-induced platelet aggregation, LOLE could directly scavenge DPPH radicals. Oral administration of LOLE also reduced the number of deaths in the intravenous injection of collagen plus epinephrine-induced mouse model of pulmonary thrombosis [66].

Secolincomolide A, a compound isolated from LO, showed platelet activity in collagen-induced platelet aggregation and serotonin secretion in platelets freshly isolated from a rabbit (Table 2). Interestingly, Secolincomolide A effectively decreased the production of diacylglycerol, arachidonic acid, thromboxane B2 (TXB2), and prostaglandin D2 (PGD2). In an arterial thrombosis model, this compound also prolonged the bleeding time and reduced FeCl3-induced thrombus formation. In addition, Secolincomolide A inhibited the activation of the collagen receptor, glycoprotein VI (GPVI) and inhibited phosphorylation of spleen tyrosine kinase (Syk) p47, phospholipase Cγ2 (PLCγ2), extracellular signal-regulated kinase 1/2 (ERK1/2) and protein kinase B (Akt). The researchers concluded that Secolincomolide A inhibits the GPVI-mediated signalling pathway and the COX-1-mediated arachidonic acid (AA) metabolism pathway [18]. Overall, both extracts and compounds isolated from LO have shown antiplatelet and antithrombotic effects in both in vitro and in vivo models through modulating different molecular pathways. Therefore, further elucidation of LO extracts and its components on different models of pulmonary thrombosis may provide potential drug candidates to treat such disorders.

### 5.3. Cytotoxic Activity

Kwon et al. (1999) reported that five compounds—Linderin A, (+)-xanthoxyol, Pluviatilol, Actiforin, 5,6-dihydroxymatairesinol, (+)-syringaresinol and (+)-9’-O-trans-feruloyl-5,5’-dimethoxylariciresinol—possessed a cytotoxic effect on a small panel of human tumour cell lines [67]. (−)-Syringaresinol, one of the constituents of LO, has been reported to exhibit cytotoxic activity. (−)-Syringaresinol significantly inhibited the human promyelocytic HL-60 leukaemia cell proliferation via G1 arrest and also induced apoptosis (Table 2). (−)-syringaresinol treatment increased the expression of Cdki proteins (p21(cip1/waf1) and p27(kip1)) and decreased the cyclin D(1), cyclin D(2), cdk2, cdk4, cdk6 and cyclin E expression, thus arresting the G(1) phase. It also induced apoptosis through fragmenting the DNA, altering the Bax/Bcl-2 ratio and cleavage of poly (ADP-ribose) polymerase. in addition, it stimulated cytochrome c release and activated caspase-3 and caspase-9 [68]. Kwon and his team isolated three new butanolides 2-(1-methoxy-11-dodecenyl)-penta-2,4-dien-4-olide, (2*Z*,3*S*,4*S*)-2-(11-dodecenylidene)-3-hydroxy-4-methyl butanolide and (2*E*,3*R*,4*R*)-2-(11-dodecenylidene)-3-hydroxy-4-methoxy-4-methyl butanolide from the stems of LO and investigated their cytotoxic effects on tumour cell lines of human origin. The compound 2-(1-methoxy-11-dodecenyl)-penta-2,4-dien-4-olide, (2*Z*,3*S*,4*S*)-2-(11-dodecenylidene)-3-hydroxy-4-methyl butanolide showed significant cytotoxicity against five different human cell lines: no-small cell lung cancer (A549), skin cancer (SK-MEL-2), CNS cancer (XF498), ovarian (SK-OV-3) and colon cancer (HCT15) [63].

### 5.4. Hepatoprotective Activity

In an in vitro study, (+)-episesamin isolated from a 70% ethanolic reaction showed antifibrotic activity. (+)-episesamin reduced the expression of profibrotic autocrine TGF-β, and therefore stopped the proliferation of hepatic stellate cell (HSC) without any significant cytotoxicity (Table 2) [69]. In another in vivo study, a 70% ethanolic extract of LO showed remarkable hepatoprotection against tert-butyl hydroperoxide-induced oxidative hepatotoxicity in rats. LO extract also prevented tert-butyl hydroperoxide (t-BHP)-induced oxidative damage in hepatic HepG2 cells. However, pre-treatment with LO extract significantly lowered the serum levels of alanine and aspartate aminotransferases, and glutathione levels were increased in the liver, decreasing the lipid peroxidation in a dose-dependent manner. The LO extract also significantly reduced t-BHP-induced hepatotoxicity [61]. In another study, LO extract decreased intracellular oxidative stress and lowered the expression of a tissue inhibitor of metalloproteinases (TIMP)-1 in activated rat and human hepatic stellate cells (HSCs). LO extract also disrupted TGF-β autoinduction and increased the expression of MMP-3, MMP-2 and gelatinolytic activity. These findings demonstrated that the antifibrotic effect of LO extract is mediated by antioxidant activity (Figure 2) [70].

### 5.5. Vasoprotective and Antihypertensive Activity

An array of evidence suggests that in type II diabetes (T2DM), dysregulation of the angiotensin system contributes to impaired endothelial function. In contrast, angiotensin-converting enzyme (ACE) inhibitors and angiotensin II (Ang II) receptor type I blockers have been used for a long time to prevent endothelial dysfunction in T2DM patients. Moreover, Ang II is a potent inducer of NADPH oxidase-derived vascular oxidative stress and endothelial dysfunction [71]. One study demonstrated the beneficial effect of LO stem extract (LOSE) on the vascular system in db/db mice. LOSE improved the capacity of physical exercise and normalized the angiotensin system and metabolic parameters through improving endothelium-dependent relaxations and vascular oxidative stress. Further, treatment with LOSE (100 mg/kg/day by gavage for eight weeks) restored the vascular oxidative stress through increasing the expression of cyclooxygenases, NADPH oxidase, angiotensin II, angiotensin type 1 receptor, and peroxynitrite. Further, LOSE treatment significantly decreased the expression of endothelial NO synthase in db/db mice in comparison with the antidiabetic drug pioglitazone (30 mg/kg/day by gavage) [50]. Interestingly, in the LOSE-administrated group, lower blood glucose level, albumin–creatinine ratio, and reduced body weight were observed. These results were due to the inhibition of purified ACE, COX-1, and COX-2. Overall, the study suggests that LOSE restores the angiotensin system and resets its metabolic parameters, therefore improving the physical performance of diabetic mice. Hence, LOSE is a potential vasoprotective agent that may be transformed into a therapeutic agent in future.

Atherosclerosis is characterized by the accumulation of thrombus, cells or lipids plaques within the arterial intima [72]. The activation of vascular smooth muscle cells (VSMC) is a major contributor to atherosclerosis and generates ROS. Increased ROS promotes acute inflammatory responses and subsequent vasculature dysfunction in atherosclerotic lesions [73]. Therefore, inhibiting or blocking the activation or proliferation of VSMC has proven to be a rational approach in the treatment of atherosclerosis [74].

The lignan (+)-episesamin, one of the active constituents of LO, has been reported to interfere with the TNF-α-induced activation of VSMC via diminishing activation of NF-ĸB, ERK1/2 and AKT and decreased activity of gelatinases. Activation of VSMC is the key event in the pathogenesis of atherosclerosis. VSMC is triggered by TNF-α, which results in a mitogenic VSMC response. (+)-episesamin (1–10 μM) inhibits the activation of Akt, NF-ĸB and MMP-2/-9, thus inhibiting TNF-α-induced proliferation of human and murine VSMC. Moreover, (+)-episesamin reduced TNF-α- and H_2_O_2_ induced oxidative stress through increasing HO-1 expression [16]. Overall, the study showed that (+)-episesamin decreases VSMC activation, proliferation, and migration, and therefore contributes to the formation of atherogenesis. The strong antioxidant property of (+)-episesamin suggests that it has potential for the treatment of VSMC activation-associated vascular diseases, such as atherosclerosis, hypertension, and cardiovascular disorders.

### 5.6. Anti-Melanogenic Activities

The study suggested the antioxidant and whitening effects of LO on B16 melanoma F10 cells. 70% EtOH extracts of the leaf and branch of LO dose-dependently scavenged DPPH, hydroxyl, and superoxide anion radicals. Moreover, leaf extracts demonstrated ERK pathway activation and downregulation of MITF and tyrosinase and, therefore, a decrease in melanogenesis in B16 melanoma F10 cells [51]. Hong et al. (2013) isolated quercitrin (quercetin-3-O-α-l-rhamnopyranoside) and afzelin (kaempferol-3-O-α-l-rhamnoside) from the ethyl acetate fraction of LO and evaluated their antimelanogenic effect on melanoma cells. Both compounds showed significant antioxidant activities in a DPPH radical scavenging assay and FRAP assay as well as antimelanogenic activities through an inhibiting tyrosinase activity. In contrast, quercitrin modulated the ERK and MITF signalling pathway in B16F10 melanoma cells [17].

### 5.7. Neuroprotective Activity

Lignans display significant neuroprotective activities against glutamate-induced toxicity in primary cultures of rat cortical cells [75]. Lee et al. (2010) isolated two new secoisolariciresinol derivatives 9,9’-o-di-(e)-feruloyl-meso-5,5’-dimethoxysecoisolariciresinol (a) and 9,9’-O-di-(e)-sinapinoyl-meso-5,5’-dimethoxysecoisolariciresinol (b) with a known compound 9,9’-O-di-(e)-feruloyl-meso-secoisolariciresinol(c) isolated from the methanolic extract of LO stems (LOSE). Among these three compounds, (a) and (c) showed significant neuroprotective activity against glutamate-induced oxidative stress in HT22 hippocampal cells in a 3-(4,5-dimethylthiazol-2-yl)-2,5-diphenyltetrazolium bromide (MTT) assay [76]. However, because the neuroprotective activity was claimed based on an MTT assay alone, further study is required to confirm this neuroprotective activity.

## 6. Conclusions

Plants are the primary source of bioactive compounds that can be directly used for drugs or further modified into therapeutic agents. Both extracts and isolated bioactive compounds of LO have been reported to be effective in many oxidative stress-associated diseases. Most of these compounds possess remarkable in vitro and in vivo antioxidant and other pharmacological properties through the modulation of different inflammatory pathways (NF-κB, TNF-α, MAPK), cell proliferation (Cyclin D, E; Gi phase), antioxidative (Nrf-2/HO-1), apoptosis (Bax/Bcl-2, Cas-3), antimelanogenic (MITF), etc., but further research is necessary to explore the specific cellular and molecular targets of all of these active constituents. A detailed investigation is also required to study the mechanism of actions of potentially bioactive compounds such as (+)-syringaresinol, quercitrin, afzelin and (+)-episesamin for their diverse role in inflammation, cell proliferation and allergy. For example, several compounds have been discovered that possess neuroprotective potential; however, this claim requires an in-depth investigation. Despite ethanolic, methanolic and water extract of the different parts of LO yielding numerous bioactive constituents, most of these compounds remain uninvestigated for their pharmacological activities. Such investigations may provide new lead compounds for the development of future therapeutic agents. 

## Figures and Tables

**Table 1:** Antioxidant properties of *Lindera genus* plants.

Species	Extract/Compound	Plant Part	Model	Tested Concentrations	IC_50_	Major Effects	Ref.
***L. aggregata***	Water and EtOH	root	alcoholic liver injury	2 g/kg	-	decreased MDA and SOD levels	[21]
***L. aggregata***	75% EtOH	root		1, 2, and 4 g/kg	-	suppressed TLR4 overexpression and promoted the expression of occludin and claudin-1 in intestine tissue	[47]
***L. aggregata***	EtOH	leaves	DPPH free radical scavenging assay	-	High Contents total phenols (May collection) 542.4 ± 12.9 μg/mL	Higher major compound (quercetin-3-O-α-l-rhamnoside)	[22]
***L. aggregata***	Quercetin-3-O-α-l-rhamnopyranoside	leaves	HUVEC cells	62.5, 125, 250, and 500 µM	-	promoting Nrf2 and HO-1	[5]
***L. aggregata***	Lindera aggredin C	whole plants	Human Neutrophils	-	7.45 ± 0.74 µM	inhibition of superoxide anion generation	[23]
	(+)-*N*-methyllaurotetanine			-	8.36 ± 0.11 µM		
	(+)-isoboldine			-	5.81 ± 0.59 µM		
***L. aggregata***	linderane	root	INS-1 cells	5, 10, and 20 µM		activation of Nrf2 pathway	[48]
***L. aggregata***	Linderagalactone E,	root	HepG2 cells	-	67.5 µM	hepatoprotective activity	[25]
	linderane			-	167.0 µM		
	hydroxylindestenolide			-	42.4 µM		
	linderalactone			-	98.0 µM		
***L. aggregata***	quercitrin	leaves	HCL mouse models	0.3, 0.6, and 1.2 g/kg	-	combine Keap-1/Nrf2 system	[29]
***L. aggregata***	water	leaves	In vitro assay	-	1.0 mg/mL (Hydroxyl radical)	scavenging activity of ROS and RNS, and inhibits lipid peroxidation	[6]
				-	0.01 mg/mL (Superoxide anion radical)		
				-	approximately 2 to 3 mg/mL (NO generation		
				-	0.08 mg/mL (lipid peroxidation)		
***Lindera aggregata***	Lindenenyl acetate	root	HT22 cells	10, 20, 30, and 40 µM	-	increasing the activity of HO	[31]
***Lindera aggregata***	water	roots	Post-ischemic Myocardial rats	0.75 and 1.5 g/kg	-	scavenging activities on DPPH radical	[30]
***L. erythrocarpa***	Lucidone	fruit	HaCaT cells	0.5, 1, 5, and 10 µg/mL	-	increased expression HO-1	[33]
***L. erythrocarpa***	Lucidone	fruit	Ava5 cells	5, 10, 20, 30, 40, and 50µM	-	increased gene expression of HO-1 or Nrf2	[34]
***L. erythrocarpa***	(−)-epicatechin	aerial parts	H9c2 cells	-	1.7 µM	reduced the release of LDH	[28]
	avicularin			-	0.7 µM		
	quercitrin			-	22.3 µM		
***L. pulcherrima***	Extraction of oils	leaf	In vitro assay	-	0.087 ± 0.03 mg/mL	DPPH radical scavenging activity	[7,35]
***L. glauca***	Water	Stem	In vitro assay	-	11.920 ± 0.213 µg/mL (DPPH radical)	Free radical scavenging activity	[36]
				-	54.348 ± 2.124 (Alkyl radical)		
				-	4.436 ± 0.141 (hydroxyl radical)		
	EtOH			-	13.357 ± 0.312 µg/mL (DPPH)		
				-	56.714 ± 2.223		
				-	2.868 ± 0.124		
***L. glauca***	EtOH	Stem	In vitro assay	-	30.5 ± 0.6 µg/mL	scavenging activities on DPPH radical	[37]
		root		-	29.4 ± 0.3 µg/mL		
***L. neesiana***	Water	fruit	N2a cells	10, 50, and 100 µg/mL	-	increased Nrf2 secretion	[39]
***L. neesiana***	60% EtOH	leaves and twigs	In vitro assay	-	20.9 ± 1.04 µg/mL	DPPH radical scavenging activity	[40]
***L. oxyphylla***	Flavokawain B	bark	In vitro assay	-	8.5 ± 0.004 μg/mL	DPPH radical scavenging activity,	[45]
***L. reflexa***	Pinosylvin	root	ARPE-19 cells	5 µM		increase of HO-1	[42,49]
***L. umbellata***	(2S,3S)-2,3-bis[(4-hydroxy-3,5-dimethoxyphenyl)methyl]-1,4-butanediol 1,4-diferulate	Stem twig	In vitro assay	-	22.5 ± 1.8 μg/mL	DPPH radical scavenging activity,	[46]
	ssioriside				21.5 ± 2.1 μg/mL		
	lyoniside				26.3 ± 0.5 μg/mL		
	(+)-9′-O-(E)-feruloyl-5,5′-dimethoxylariciresinol				23.6 ± 1.9 μg/mL		
***L. obtusiloba***	MeOH extract	leaves	NCI-H292 cell	25, 50, and 100 uM	-	increased the translocation of Nrf-2 into the nucleus with elevated HO-1 expression	[15]
***L. obtusiloba***	EtOH	stem	In vivo: type 2 diabetes mellitus mice model	100 mg/kg	-	expression of the NADPH oxidase subunits NOX-1 and p47phox	[50]
***L. obtusiloba***	70% EtOH extract	leaves	t-BHP rat model	500 and 2000 mg/kg	-	decreased GSH level and oxidized NADPH	
			In vitro assay	-	249.5 ± 1.9 µg/mL	DPPH radical scavenging activity	
	Quercitrin			-	6.9 ± 0.4		
	Afzelin			-	47.3 ± 2.4		
***L. obtusiloba***	MeOH extract	leaves	In vitro assay		4.21 ± 0.09 µg/mL	DPPH radical scavenging activity	
	Quercitrin				107.5 ± 4.1 µM		
	Afzelin				438.7 ± 14.2 µM		
***L. obtusiloba***	70% EtOH extract	leaves	In vitro assay	-	243.14 µg/mL (DPPH)	DPPH, superoxide radical and hydroxyl radical scavenging activity	[51]
					35.47 µg/mL (enzymatic system of superoxide radical assay)		
					1.21 mg/mL (Hydroxyl radical)		
		branch/stem mixed			181.10 µg/mL (DPPH)		
					>100 µg/mL (enzymatic system of superoxide radical assay)		

**Table 2:** Selective bioactive compounds of *Lindera obtusiloba* and their pharmacological activities.

Compounds	Plant Part	Extraction Method	Study Model/Dose	Main Findings	Activity	Ref.
**Anti-Allergic Inflammatory Activities**						
**Isotachioside**, **Koaburaside**, **2,6-dimethoxy-4-hydroxyphenyl-1-O-ß-d-glucopyranoside**, **4,6-dihydroxy-2- methoxyphenyl-1-O-ß-d-glucopyranoside**, **Erigeside C**, **Salidroside**, **6-hydroxyphenyl)-1-O-ß-d-glucopyranoside**	Stem	Methanol	In vitro HMC-1 cells (10 µM)	Inhibited histamine release in mast cells. Hydroxyphenyl)-1-O-ß-d-glucopyranoside significantly inhibited in histamine release and IL-6 and TNF-α production in mast cells	Anti-allergic inflammatory activities	[60]
**(+)-(7*S*,8*R*,80*R*)-acuminatolide**, **(+)-9′-*0*-O-trans-feruloyl-5,5–dimethoxylariciresinol**	Stem	Methanol	In vitro HMC-1 cells (10 µM)	inhibited histamine release	Anti-allergic activity	
**(+)-syringaresinol**	Stem	Methanol	In vitro RAW 264.7 cells (25, 50, and 100 μM)	suppressed iNOS, COX-2, TNF-α, IL-1β, and IL-6 mRNA levels as well as COX-2 and NF-κB protein levels	Anti-inflammatory activity	[65]
			In vivo Male ICR mice (30 mg/kg)	suppressed carrageenan-induced elevation of iNOS, COX-2, TNF-α, IL-1β, and IL-6 mRNA levels as well as COX-2 and NF-κB protein levels	Anti-inflammatory activity	
**(+)-Episesamin**	Twigs	70% Ethanol	In vitro Hepatic stellate cells (10, 20, 50 µM) MOVAS-1 cell line (10 µM)	blocked cell proliferation and the profibrotic autocrine TGF-β expression HSC without significant cytotoxicity reduced TNF-α- and H_2_O_2_ -induced oxidative stress and in parallel induces anti-inflammatory haem oxygenase (HO)-1 expression	Antioxidant, Anti-inflammatory and other activities	[16]
**Antiplatelet**						
**Asarinin**, **Secoisolitsealiicolide B,** **Secolincomolide A**	Stem	Methanol	In vivo Male white rabbits	inhibited of the GPVI and the COX-1-mediated metabolic pathways	Antiplatelet activity	[18]
**Cytotoxicity**						
**Linderin A**, **(+)-xanthoxyol**	Stem	Methanol	In vitro HMC-1 cells (10 µM	inhibited histamine release and production of IL-6 and TNF-α.	Cytotoxicity and inflammatory activity	[62,67]
**Pluviatilol**			In vitro Tumour cells (3.40–19.27 µg/mL)	blocked cell proliferation of human tumour cell lines	Cytotoxicity	
**Actiforin**			In vitro Tumour cells (3.40–19.27 µg/mL) HMC-1 cells (10 µM)	blocked cell proliferation of human tumour cell lines inhibited the histamine release and production of IL-6 and TNF-α.	Cytotoxicity and anti-inflammatory activity	
**5,6-dihydroxymatairesinol**, **(+)-syringaresinol**, **(+)-9’-O-trans-feruloyl-5,5′-dimethoxylariciresinol**, **2-(1-methoxy-11-dodecenyl)-penta-2,4-dien-4-olide**, **(2*Z*,3*S*,4*S*)-2-(11-dodecenylidene)-3-hydroxy-4-methyl butanolide**, **(2*E*,3*R*,4*R*)-2-(11-dodecenylidene)-3-hydroxy-4-methoxy-4-methyl butanolide**, **(−)-syringaresinol**	Stem	Methanol	In vitro Tumour cells (3.40–19.27 µM)	blocked cell proliferation of human tumour cell lines	Cytotoxicity	[63]
**Neuroprotective**						
**9,9′-O-di-(E)-feruloyl-meso-5,5′-dimethoxysecoisolariciresinol**, **9,9′-O-di-(E)-sinapinoyl-meso-5,5′-dimethoxysecoisolariciresinol**, **9,9′-O-di-(E)-feruloyl-meso-secoisolariciresinol**	Stem	80% Methanol	In vitro HT22 cells (1.0 and 10 µM)	protected from glutamate induced neurotoxicity in HT22 cells	Neuroprotective activity	[76]
**Antiatherosclerosis**						
**(+)-episesamin**	Fruit	-	In vitro Human and murine VSMC	Diminished the activation of NF-ĸB, ERK1/2 and AKT and decreased activity of gelatinases inhibited the activation of Akt, NF-ĸB and MMP-2/-9 thus inhibiting TNF-α-induced proliferation of VSMC	Antiatherosclerosis Antioxidant activity	[16]
**Hepatoprotective**						
**(+)-episesamin**	Twigs	70% Ethanol	In vitro Hepatic stellate cells (10, 20, and 50 µM)	blocked the proliferation and the profibrotic autocrine TGF-β expression HSC without significant cytotoxicity	Hepatoprotective activity	[69,70]
**Antimelanogenic**						
**Quercetin-3-O-α-l-rhamnopyranoside**, **Kaempferol-3-O-α-l-rhamnoside**	Leaves	Methanol	In vitro B16F10 melanoma cells (100 and 150 µM)	modulates of ERK and MITF expression	Antioxidant and antimelanogenic activity	[17]

## Abbreviations

ROS, reactive oxygen species; SOD, superoxide dismutase; GPx, glutathione peroxidase; 2,2-dipheny-l-picrylhydrazyl; ABTS, 2,2′-azinobis-3-ethylbenzothiazoline-6-sulphonate; MDA, malondialdehyde; GSH, glutathione; GST, glutathione-S-transferase; SOD1, Zn-superoxide dismutase; NF-κB, nuclear factor kappa B; LPS, lipopolysaccharide; NO, nitric oxide; TNF-α, tumour necrosis factor-alpha; IL-6, interleukin-6; PGD2, prostaglandin D2; PGE2, prostaglandin E2; IL-1β, interleukin-1 β; iNOS, inducible nitric oxide synthase; COX-2, cyclooxygenase-2; MAPK, mitogen-activated protein kinase; TH, tyrosine hydroxylase; LDH, lactose dehydrogenase; MTT, 3-(4,5-dimethylthiazol-2-yl)-2,5-diphenyltetrazolium bromide; hepatocellular carcinoma cells; ERK, extracellular receptor kinase; AKT, reduced protein kinase B; ARE, antioxidant response elements; Nrf2, phospho-nuclear factor erythroid 2-related factor; HO-1, hemoxygenase; alanine amino transferase; AST, aspartate amino transferase; LDH, lactate dehydrogenase; FRAP, ferric reducing antioxidant power; HSC, hepatic stellate cell; VSMC, vascular smooth muscle cells TXA2, thromboxane A2; aPTT, partial thromboplastin time; PT, prothrombin time; PLCγ2, phospholipase Cγ2; Syk, spleen tyrosine kinase; Akt, protein kinase B; GPVI), glycoprotein VI; LDL, low-density lipoprotein; AA, arachidonic acid; TIMP1, tissue inhibitor of metalloproteinases; HaCaT, human keratinocyte.
